# Effect of passive exposure to cigarette smoke on blood pressure in children and adolescents: a meta-analysis of epidemiologic studies

**DOI:** 10.1186/s12887-019-1506-7

**Published:** 2019-05-21

**Authors:** Mahshid Aryanpur, Mahmoud Yousefifard, Alireza Oraii, Gholamreza Heydari, Mehdi Kazempour-Dizaji, Hooman Sharifi, Mostafa Hosseini, Hamidreza Jamaati

**Affiliations:** 1 Tobacco Prevention and Control Research Center, National Research Institute of Tuberculosis and Lung Diseases (NRITLD), Shahid Beheshti University of Medical Sciences, Tehran, Iran; 2 Physiology Research Center, Faculty of Medicine, Iran University of Medical Sciences, Tehran, Iran; 3 Department of Medicine, Tehran University of Medical Sciences, Tehran, Iran; 4 Mycobacteriology Research Center, Biostatistics Unit, NRITLD, Shahid Beheshti University of Medical Sciences, Tehran, Iran; 5 Department of Epidemiology and Biostatistics, School of Public Health, Tehran University of Medical Sciences, Poursina Ave, Tehran, Iran

**Keywords:** Hypertension, Blood pressure, Children and adolescent, Smoking

## Abstract

**Background:**

Hypertension is an emerging disease in children and adolescents resulting in future morbidities. Cigarette smoking is one of the most studied contributing factors in this regard; however, there are contradictory results among different studies. Therefore, the present meta-analysis tends to assess the relationship between passive exposure to cigarette smoke and blood pressure in children and adolescents.

**Method:**

Medline, Embase, Scopus, EBSCO, and Web of Sciences were systematically reviewed for observational studies up to May, 2017, in which the relationship between cigarette smoking and hypertension were assessed in children and adolescents. The meta-analysis was performed with a fixed effect or random effects model according to the heterogeneity.

**Results:**

Twenty-nine studies were included in present meta-analysis incorporating 192,067 children and adolescents. Active smoking (pooled OR = 0.92; 95% CI: 0.79 to 1.05) or passive exposure to cigarette smoke (pooled OR = 1.01; 95% CI: 0.93 to 1.10) were not associated with developing hypertension in the study population. Despite the fact that active cigarette smoking did not significantly affect absolute level of systolic and diastolic blood pressure, it was shown that passive exposure to cigarette smoke leads to a significant increase in absolute level of systolic blood pressure (pooled coefficient = 0.26; 95% CI: 0.12 to 0.39).

**Conclusion:**

Both active and passive cigarette smoking were not associated with developing hypertension in children and adolescents. However, passive cigarette smoke was associated with higher level of systolic blood pressure in children and adolescents.

## Background

Hypertension has been named “Silent Killer” by some researchers as it is a disease that can lead to cardiovascular disorders, cerebral infarction and renal failure [1]. About 1–3% of children have hypertension [2] which has a secondary etiology in about 80% of cases and is a consequence of an underlying factor such as family history, body mass index, socioeconomic status and nutritional status [3–5]. Some studies have reported that cigarette smoking is a risk factor for hypertension. There are strong evidence that exposure to cigarette smoke has adverse effects on health during childhood, adolescence and even adulthood [6–8]. Studies show that children exposed to cigarette smoke during fetal life have significantly lower birth weights in addition to higher risk of getting overweight or obese in future [9]. Moreover, active smoking or passive exposure to cigarette smoke cause dysfunction of capillary endothelium in healthy individuals suggesting an association between cigarette smoking and hypertension [10]. However, some studies report that there is no association between cigarette smoking and hypertension in children [11].

The importance of this issue is that both cigarette smoking and hypertension are two risk factors of non-communicable diseases [12, 13]. Therefore, presence of two risk factors in a single individual may lead to an additive or synergistic effect on incidence of chronic diseases. This issue must be more emphasized in childhood as most diseases of adulthood are consequences of childhood health status.

Multiple studies have been conducted regarding the association between exposure to cigarette smoke and hypertension in recent years in the field of pediatrics. However, contradictory results were reported in various studies. Hence, the present meta-analysis was designed to assess the association between exposure to cigarette smoke and systolic and diastolic blood pressure in addition to its risk for incidence of hypertension in children and adolescents.

## Methods

### Study design

The present study is designed based on instructions of Meta-analysis of Observational Studies in Epidemiology (MOOSE) statement [14]. All cohort, case-control and cross sectional studies on children and adolescents between the ages of 0 and 18 years old assessing the relation of exposure to cigarette smoke and hypertension were reviewed. Exclusion criteria were combination of results with data of adults, lack of adjustment for potential confounders, review articles and lack of reported odds ratio (OR) or regression coefficient (Beta).

### Search strategy

In the present study, an extensive search was performed in electronic databases of Medline (via PubMed), Embase, Scopus, EBSCO, and Web of Sciences until the end of May, 2017. Keywords were selected using databases of Mesh and Emtree and with the help of specialists in fields of hypertension and cigarette smoking. These keywords were phrases related to usage or exposure to cigarette smoke and hypertension. Search query in Medline is shown in Table 1. In addition, a manual search was done in the bibliography of related articles, contact was made with authors of related articles and at the end a search in the thesis division of the ProQuest database to screen additional articles and unpublished data. Additionally, Google search engine and Google scholar were also used to find Grey literature.

**Table 1:** Search strategy of present study in Medline

Databases	Search query
Medline (via PubMed)	((((“Smoking”[Mesh] OR “Tobacco”[Mesh] OR “Tobacco Use”[Mesh] OR “Smoking”[tiab] OR “Tobacco”[tiab] OR “Tobacco Use”[tiab] OR “Cigar Smoking”[tiab] OR “Smoking, Cigar”[tiab] OR “Tobacco Smoking”[tiab] OR “Smoking, Tobacco”[tiab] OR “Hookah Smoking”[tiab] OR “Smoking, Hookah”[tiab] OR “Waterpipe Smoking”[tiab] OR “Smoking, Waterpipe”[tiab] OR “Pipe Smoking”[tiab] OR “Smoking, Pipe”[tiab] OR “Cigarette Smoking”[tiab] OR “Smoking, Cigarette”[tiab] OR “Tobaccos”[tiab] OR “Tobacco Uses”[tiab] OR “Tobacco Consumption”[tiab] OR “Consumption, Tobacco”[tiab] OR “Cigars”[tiab] OR “Cigar”[tiab] OR “Cigarettes”[tiab] OR “Cigarette”[tiab] OR “second hand smoke”[tiab] OR “secondhand smoke”[tiab] OR “second-hand smoke”[tiab] OR “passive smoking”[tiab] OR “tobacco consumption”[tiab] OR “cigarette smoke”[tiab] OR “tobacco consumption”[tiab])) AND (“Hypertension”[Mesh] OR “Blood Pressure”[Mesh] OR “Arterial Pressure”[Mesh] OR “Hypertension”[tiab] OR “Blood Pressure”[tiab] OR “Arterial Pressure”[tiab] OR “Arterial Pressures”[tiab] OR “Pressure, Arterial”[tiab] OR “Pressures, Arterial”[tiab] OR “Arterial Tension”[tiab] OR “Arterial Tensions”[tiab] OR “Tension, Arterial”[tiab] OR “Tensions, Arterial”[tiab] OR “Blood Pressure, Arterial”[tiab] OR “Arterial Blood Pressure”[tiab] OR “Arterial Blood Pressures”[tiab] OR “Blood Pressures, Arterial”[tiab] OR “Pressure, Arterial Blood”[tiab] OR “Pressures, Arterial Blood”[tiab] OR “Mean Arterial Pressure”[tiab] OR “Arterial Pressure, Mean”[tiab] OR “Arterial Pressures, Mean”[tiab] OR “Mean Arterial Pressures”[tiab] OR “Pressure, Mean Arterial”[tiab] OR “Pressures, Mean Arterial”[tiab] OR “Pressure, Blood”[tiab] OR “Diastolic Pressure”[tiab] OR “Pressure, Diastolic”[tiab] OR “Pulse Pressure”[tiab] OR “Pressure, Pulse”[tiab] OR “Systolic Pressure”[tiab] OR “Pressure, Systolic”[tiab] OR “Pressures, Systolic”[tiab] OR “Blood Pressure, High”[tiab] OR “Blood Pressures, High”[tiab] OR “High Blood Pressure”[tiab] OR “High Blood Pressures”[tiab] OR “Elevated Blood Pressure”[tiab] OR “Hypertensive”[tiab]))) AND (“Pediatrics”[Mesh] OR “Child”[Mesh] OR “Adolescent”[Mesh] OR “Pediatrics”[tiab] OR “Pediatrics”[tiab] OR “Paediatrics”[tiab] OR “Paediatric”[tiab] OR “Child”[tiab] OR “Adolescent”[tiab] OR “Children”[tiab] OR “Adolescents”[tiab] OR “Adolescence”[tiab] OR “Teens”[tiab] OR “Teen”[tiab] OR “Teenagers”[tiab] OR “Teenager”[tiab] OR “Youth”[tiab] OR “Youths”[tiab])

### Data extraction and quality assessment

Data extraction method is reported in our previous meta-analyses [15–24]. Search records were pooled and the duplicated studies were removed using EndNote software (version X5, Thomson Reuters, 2011). Two independent researchers screened titles and abstracts and potentially relevant studies were reviewed more precisely. Any disagreement was resolved by discussion with a third reviewer. Relevant studies were summarized including their data regarding study design, population characteristics (age and sex), sample size, outcome (hypertension, levels of systolic and diastolic blood pressure), blinding status, data gathering method (consecutive, convenience), study design (cohort, cross sectional or case-control) and possible bias. The data gathering form was designed based on instructions of PRISMA statement [25].

In the present study, two separate experiments were entered in the study if data were differentiated by sex. When regression models with different adjustments were reported, the analysis with highest number of adjustments was entered. In addition, if results were shown in graphs, the methods proposed by Sistrom and Mergo for data extraction from graphs were used [26].

At the end, study quality assessment was done using suggested instructions of Newcastle-Ottawa Scale [27]. Hence, quality of different studies was assessed based on following criteria: 1) Is the case definition adequate, 2) Representativeness of the cases, 3) Definition of controls, 4) Comparability, 5) Ascertainment of exposure, 6) Same method ascertainment case control and 7) Reporting Non-Response rate.

### Statistical analyses

Data were analysed by STATA 14.0. Analyses were done in two steps. In first step, the association between active smoking and passive exposure to cigarette smoke with hypertension in childhood and adolescence were assessed. Only studies were entered in this step which had defined hypertension as systolic or diastolic blood pressure more than 95 percentile. Hence, data were entered as adjusted OR and 95% confidence interval (95% CI).

In second step, the association between active smoking and passive exposure to cigarette smoke with absolute value of systolic and diastolic blood pressure were assessed. The related data for mentioned analysis were entered as adjusted regression coefficient (Beta) and 95% CI. The association between active smoking and hypertension was reported separately from passive exposure in all analyses. Additionally, the association between active and passive smoking with blood pressure was reported for systolic and diastolic blood pressure, separately.

Data were pooled in all analyses and an overall effect size and 95% CI were reported. Heterogeneity among studies was assessed using I^2^ test (I^2^ greater than 50% or *p* value less than 0.1 were defined as heterogeneous). Fixed effect method was used in homogenous studies and random effect model was used in case of heterogeneous studies. Subgroup analyses were done to find the source of heterogeneity which included type of study (cohort, cross sectional), age group of children under study, definition of smoker, exposure period (before birth or domestic use), parental smoking habit (mother, father and both) and sample size (less than 1000 patients and equal or greater than 1000). In addition, Egger’s test was used to assess publication bias. A *p* value of less than 0.05 was defined significant in all analyses.

## Results

### Characteristics of entered studies

**Fig. 1:**
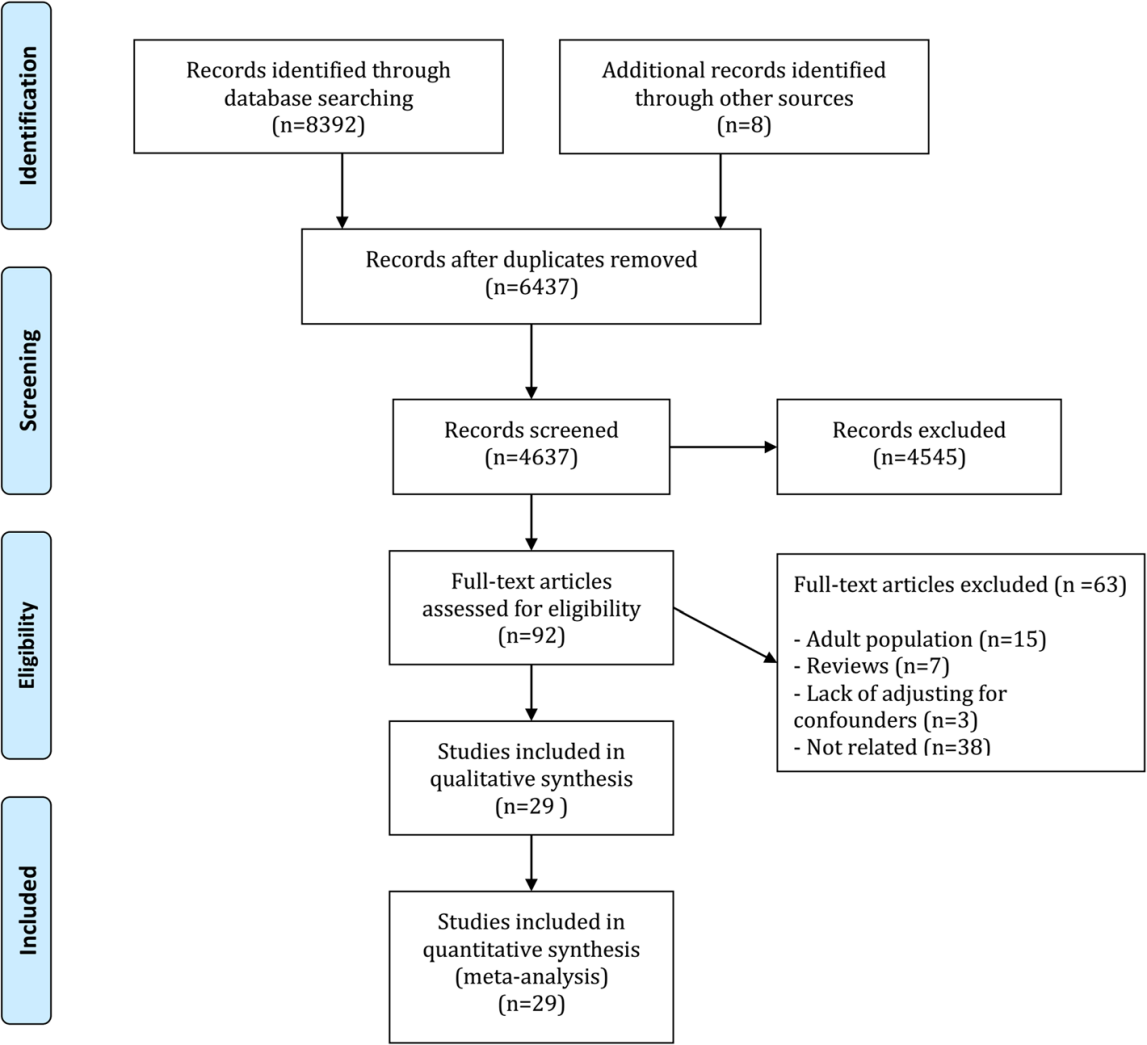
Flowchart of present meta-analysis

Eight thousand three hundred ninety-two records were gathered in the primary search. After omitting the duplicated articles and primary screening, 92 potentially relevant studies were found. At the end, 29 articles were entered in the present study after assessing their full texts [28–57] (Fig. 1). Data of 192,067 children and adolescence between the ages of 3 and 18 years old were assessed. Boys comprised 75.77% of patients. 12 cohorts, 16 cross-sectional and 1 case-control studies were entered.

Fifteen studies evaluated the association between active smoking or passive exposure to cigarette smoke with hypertension [28–42] and 17 studies assessed the association between cigarette smoking and absolute levels of systolic and diastolic blood pressure [34, 41–56]. Three of the mentioned studies assessed both types of outcome [34, 41, 42]. One of these studies was in Portuguese [35] and another one was in Korean [47].

Fifteen studies assessed the association between active smokers [28–31, 33, 35–39, 47, 48, 50, 51, 56] and 16 studies assessed the association between passive exposure to cigarette smoke [32, 34, 37, 40–46, 49, 52–56] and hypertension or absolute levels of blood pressure. Two studies assessed both types of exposure [37, 56]. 13 studies assessed the exposure during pregnancy [37, 40–44, 46, 49, 52–56], 2 studies assessed domestic exposure (after pregnancy) [32, 34] and 3 studies assessed both mentioned passive exposures [41, 42, 56].

There were different definitions of smoking among studies and in 7 studies there was no standard definition for smoker. In 7 studies being a smoker was only asked and answered with a yes/no question [35, 38, 40, 41, 45, 50, 54]. In 11 studies, the individuals were asked if they were current smoker or if they have smoked during pregnancy [31–33, 39, 42–44, 46, 47, 52, 53]. Table 2 and Table 3 show characteristics of entered studies.

**Table 2:** Summery of included studies which reported the relationship of pediatric hypertension (HTN) and smoking

Author, year; country	Type of survey	Study type	Total sample	Sex^a^	Age	HTN definition^b^	Smoking definition	Type of exposer	BP measurements method
Akis, 2009; Turkey [28]	Local	Case-control	236	42	12 to 14	BP > 95th	More than 1 cigarette per week	Active	Three times measurement of BP using an automatic sphygmomanometer device
Bozza, 2016; Brazil [29]	Local	Cross-section	1242	596	11 to 17	BP > 95th	Cigarettes smoked 10 to 30 days	Active	Two times measurement of BP using auscultatory method
Christofaro, 2015; Brazil [30]	Local	Cross-section	1231	NR	14 to 17	BP > 95th	Current daily smoking at least 1 cigarette	Active	two times measurement of BP using an automatic oscillometric device
Cinteza, 2013; Romania [31]	Regional	Cross-section	4886	2407	3 to 17	BP > 95th	Current smoking	Active	Three times measurement of BP. First measurement using an automatic oscillometric device and a BP mercury device for the second and the third measurement
Crispim, 2014; Brazil [32]	Local	Cross-section	276	145	2 to 4	BP > 95th	Current smoking	Passive (domestic)	Two times measurement of BP using a semi-automatic an oscillometric device
Dasgupta, 2006; Canada [33]	Local	Cohort	1267	1018	10 to 18	BP > 90th	Current smoking	Active	Three times measurement of BP using an automatic oscillometric device
Giussani, 2013; Italy [34]	Regional	Cross-section	1310	682	5 to 14	BP > 90th	At least one parent with smoking habit	Passive (domestic)	Two times measurement of BP using a aneroid sphygmomanometer device
Gomes, 2009; Brazil [35]	Local	Cross-section	1875	718	14 to 20	BP > 95th	NR	Active	Single measurement of BP using an automatic oscillometric device
Guo, 2011; China [36]	Local	Cross-section	4445	2298	5 to 18	BP > 95th	At least 1 cigarette per month	Active	Two times measurement of BP using a mercury sphygmomanometer device
International Collaborative Group, 1984; Europe [37]	International	Cohort	2704	NR	14	BP > 95th	More than 5 cigarette per week	Active; pregnancy	Three times measurement of BP using a mercury sphygmomanometer device
Nur, 2008; Turkey [38]	Local	Cross-section	1020	593	14 to 18	BP > 95th	NR	Active	Three times measurement of BP using a mercury sphygmomanometer device
Pileggi, 2005; Italy [39]	Local	Cross-section	603	284	6 to 18	BP > 95th	Current smoking	Active	Three times measurement of BP using a mercury sphygmomanometer device
Shankaran, 2006; USA [40]	Regional	Cohort	516	275	6	BP > 95th	NR	Pregnancy	Two times measurement of BP using an automatic oscillometric device
Simonetti, 2011; Germany [42]	National	Cross-section	4236	2181	4 to 7.5	BP > 95th	Current smoking	Passive (domestic)	Three times measurement of BP using an auscultatory aneroid sphygmomanometry device
van den Berg, 2013; Netherland [41]	Local	Cohort	3024	1521	5 to 6	BP > 90th	NR	Passive (domestic)	Two or three times measurement of BP using an automatic sphygmomanometer device

^a^Male sex (number of children);

^b^Hypertension (HTN) was defined as systolic or diastolic blood pressure more than 95th percentile; Prehypertension was defined as systolic or diastolic blood pressure between 90th and 95th percentiles

*BP* Blood pressure, *NA* Not applicable, *NR* Not reported

**Table 3:** Summery of included studies which reported the relationship of pediatric blood pressure and smoking

Author, year; country	Type of survey	Study type	Total sample	Sex^a^	Age	Type of BP	Smoking definition	Type of exposer	BP measurements method
Belfort, 2012; USA [43]	Local	Cohort	694	NR	6.5	SBP	Smoking during pregnancy	Pregnancy	Three times measurement of BP using an automatic oscillometric device
Blake, 2000; Australia [44]	Regional	Cohort	702	NR	6	SBP	Smoking at 18 weeks gestation	Pregnancy	Two times measurement of BP using a semi-automatic oscillometric device
Brambilla, 2015; Italy [45]	National	Cross-section	1294	NR	7 to 13	SBP and DBP	NR	Passive (domestic)	Three times measurement of BP using a manual sphygmomanometer device
Brion, 2007; UK [46]	Local	Cohort	6509	3281	7.7	SBP and DBP	Smoking at 18 weeks gestation	Pregnancy	Two times measurement of BP using an automatic oscillometric device
Byeon, 2007; South Korea [47]	Local	Cross-section	127	82	10 to 13	SBP and DBP	Current smoking	Active	Three times measurement of BP using an automatic oscillometric device
Garoufi, 2017; Greece [48]	Local	Cross-section	736	366	12 to 18	SBP and DBP	Smoking for at least 1 month	Active	Three times measurement of BP using an automatic oscillometric device
Giussani, 2013; Italy [34]	Regional	Cross-section	1310	682	5 to 14	SBP	Having one parent with smoking habit	Passive (domestic)	Two times measurement of BP using a aneroid sphygmomanometer device
Hogberg, 2012; Sweden [49]	National	Cohort	92,730	92,730	17 to 19	SBP and DBP	At least 1 cigarette per day	Pregnancy	Single measurement of BP using automatic and manual sphygmomanometer devices
Katona, 2010; Hungary [50]	Local	Cross-section	10,194	5163	16.6	SBP and DBP	NR	Active	Three times measurement of BP using an automatic oscillometric device
Kollias, 2009; Greece [51]	Local	Cross-section	1008	480	12 to 17	SBP and DBP	At least 1 cigarette per day	Active	Three times measurement of BP using an automatic oscillometric device
Lawlor, 2004; Australia [52]	Local	Cohort	3864	NR	5	SBP	Smoking at 18 weeks gestation	Pregnancy	Two times measurement of BP using an digital sphygmomanometer device
Oken, 2005; USA [53]	Local	Cohort	746	373	3	SBP	Current smoking	Pregnancy	Up to 5 times measurement of BP using an automatic oscillometric device
Rostand, 2005; USA [54]	Local	Cross-section	262	149	5	SBP	NR	Pregnancy	Single measurement of BP using a mercury sphygmomanometer device
Simonetti, 2011; Germany [42]	National	Cross-section	4236	2181	4 to 7.5	SBP and DBP	Current smoking	Pregnancy and domestic	Three times measurement of BP using an auscultatory aneroid sphygmomanometry device
van den Berg, 2013; Netherland [41]	Local	Cohort	3024	1521	5 to 6	SBP and DBP	NR	Pregnancy and domestic	Two or three times measurement of BP using an automatic sphygmomanometer device
Wen, 2011; USA [55]	National	Cohort	30,441	15,031	7	SBP	At least 1 cigarette per day	Pregnancy	Two times measurement of BP using a digital oscillometric device
Yang, 2013; Canada [56]	National	Cohort	13,889	7173	6.5	SBP and DBP	At least 1 cigarette per day	Pregnancy and domestic	Single measurement of BP using a manual sphygmomanometer device

^a^Male sex (number of children); *BP* Blood pressure, *DBP* Diastolic blood pressure, *NA* Not applicable; *NR* Not reported, *SBP* Systolic blood pressure

### Quality assessment of studies

**Fig. 2:**
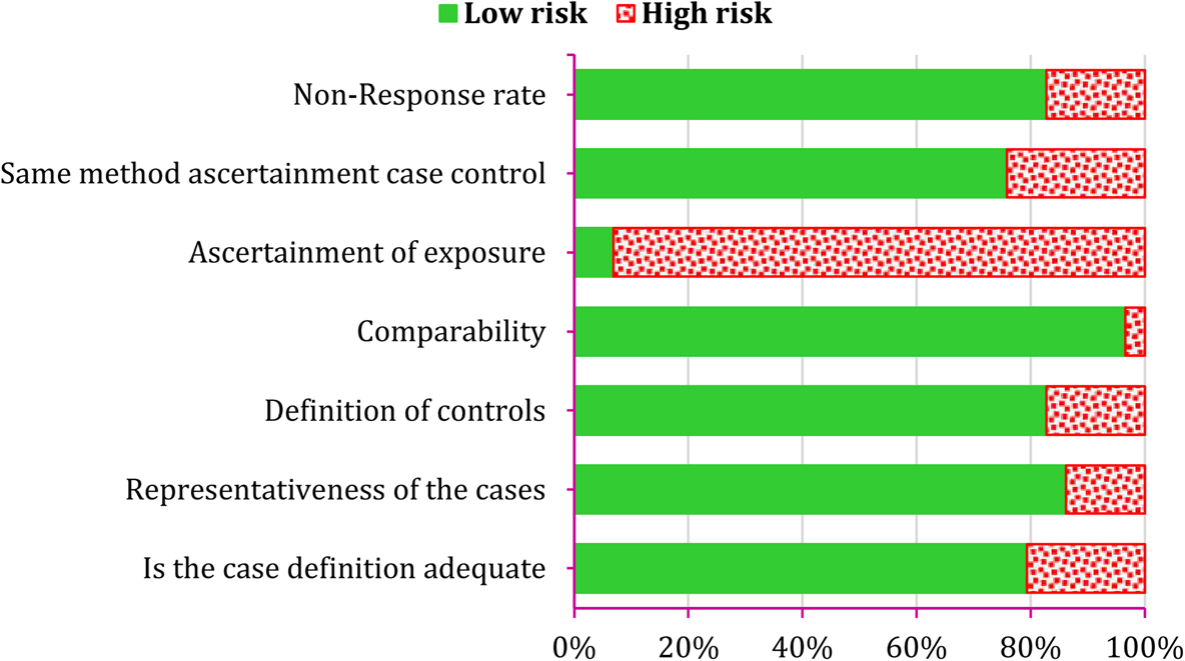
Quality assessment of included studies according to Newcastle-Ottawa Scale assessment tools

Quality assessment of studies is depicted in Fig. 2. As shown, ascertainment of exposure is biased in most studies. Other items were in appropriate levels in most studies.

### Meta-analysis

#### Effect of cigarette smoking on hypertension

### Active smoking

In the present meta-analysis, 10 studies assessed the association between active smoking and hypertension. Results were reported for boys and girls separately in the study of Dasgupta et al. [33]. Hence, the mentioned study is entered as two separate experiments. Analyses confirmed homogeneity of studies (I^2^ = 0.0%; *p* = 0.53). Additionally, publication bias was not observed in analyses (Coefficient = 1.50; *p* = 0.69).

**Fig. 3:**
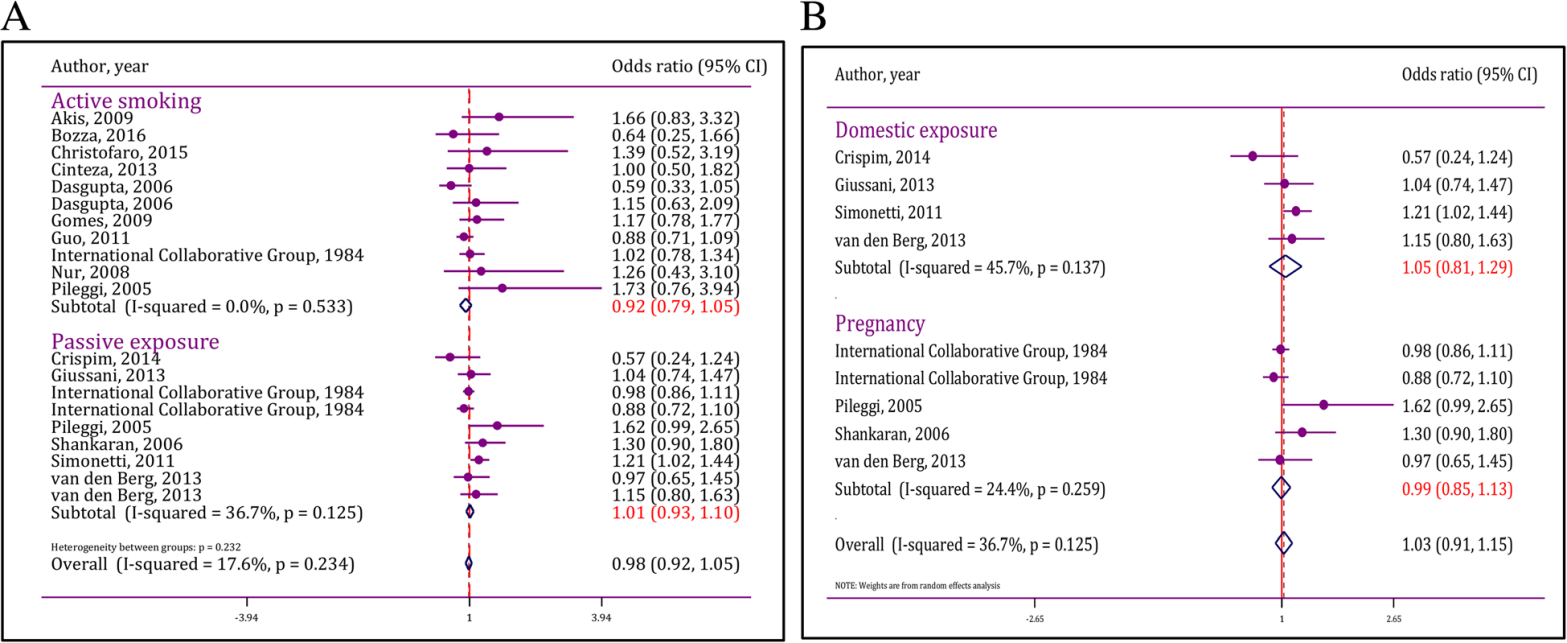
Forest plot of active and passive exposure to cigarette smoke in incidence of hypertension in children and adolescents A) Pooled odds ratio B) subgroup analysis of effect of passive exposure during pregnancy and domestic exposure on incidence of hypertension. CI: Confidence interval

Pooled analysis showed that active smoking in childhood was not associated with developing hypertension in children and adolescents (pooled OR = 0.92; 95% CI: 0.79 to 1.05). Subgroup analysis was not needed as heterogeneity was not found at this section (Fig. 3a).

### Passive exposure to cigarette smoke

7 studies were entered in order to assess the association between passive exposure to cigarette smoke and hypertension. One study assessed passive exposure in pregnancy and domestic use [37]. Hence, the mentioned study was entered in the study as two separate experiments. Heterogeneity (I^2^ = 36.7%; *p* = 0.12) and publication bias (Coefficient = 1.66; *p* = 0.80) were not present in analyses. Pooled analyses showed that passive exposure to cigarette smoke was not associated with developing hypertension in children and adolescents (pooled OR = 1.01; 95% CI: 0.93 to 1.10) (Fig. 3a).

There were two types of passive exposure to cigarette smoke among studies including exposure during pregnancy and domestic use after pregnancy. Therefore, effects of mentioned exposures were assessed separately.

### Exposure to cigarette smoke during fetal period and its association with developing hypertension

In children with passive exposure during pregnancy, exposure to cigarette smoke in fetal period did not have a significant effect on hypertension in childhood and adolescence (OR = 0.99; 95% CI: 0.85 to 1.13). Results of this section are depicted in Fig. 3b. As shown, heterogeneity (I^2^ = 24.4%; *p* = 0.26) and publication bias (Coefficient = 3.50; *p* = 0.61) were not observed.

### Effect of domestic exposure to cigarette smoke on hypertension

It was shown that domestic exposure (after fetal period) to cigarette smoke was not associated with developing hypertension (OR = 1.05; 95% CI: 0.81 to 1.29). Additionally, heterogeneity (I^2^ = 45.7%; *p* = 0.14) and publication bias (coefficient = − 15.8; *p* = 0.29) was not observed in this section (Fig. 3b).

#### Effect of cigarette smoking on absolute level of systolic and diastolic blood pressure

### Effect of active cigarette smoking on level of systolic blood pressure

**Fig. 4:**
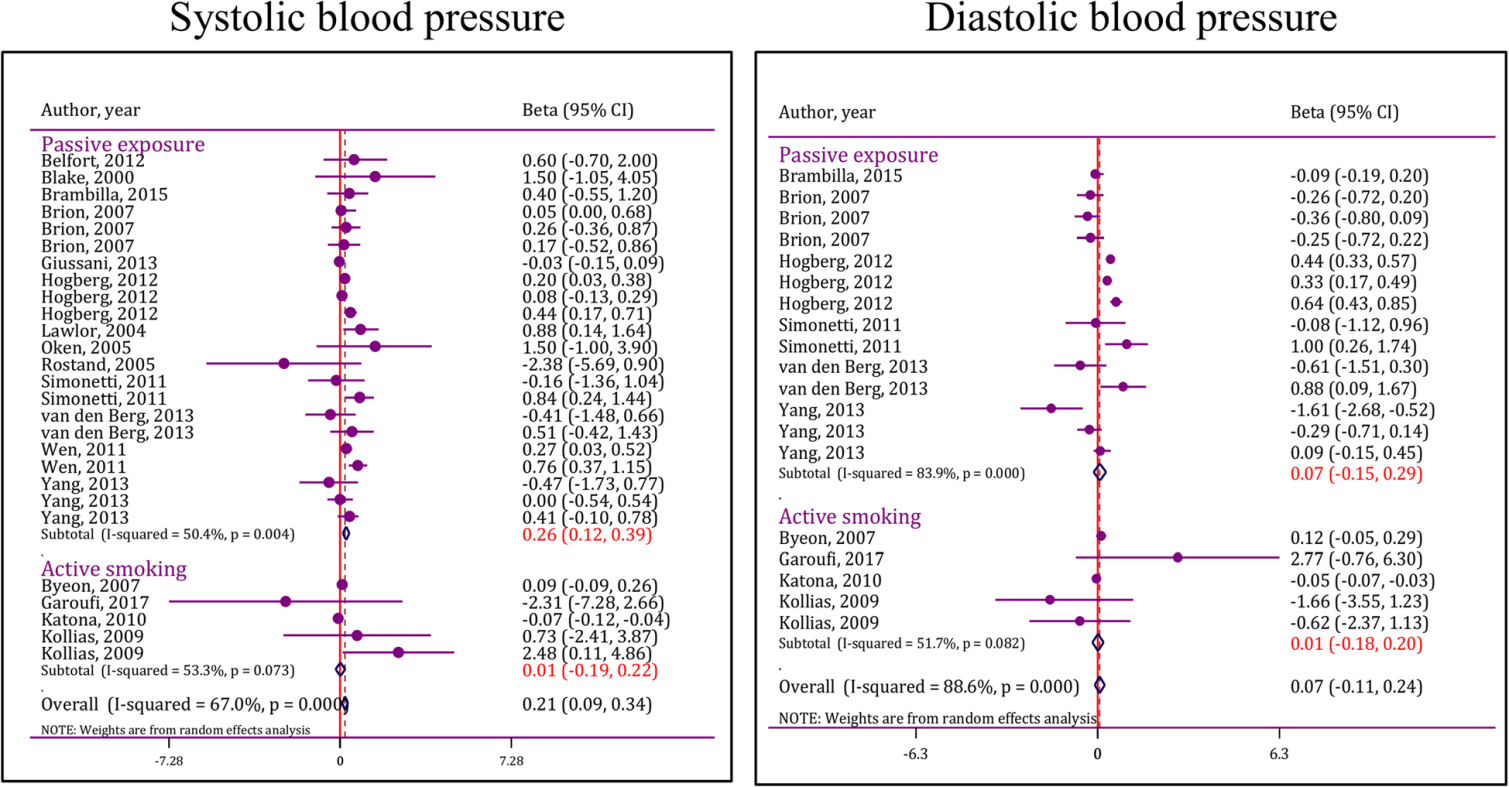
Forest plot of effect of active and passive exposure to cigarette smoke on absolute level of systolic and diastolic blood pressure. CI: Confidence interval

Results of this section are depicted in Fig. 4. Analyses in this section were done based on random effect model due to heterogeneity among studies (I2 = 53.3%; *p* = 0.07). At the end, it was shown that active cigarette smoking does not significantly affect absolute level of systolic blood pressure (pooled Beta = 0.01; 95% CI: -0.19 to 0.22). Publication bias was not observed in this section (coefficient = 5.21; *p* = 0.38).

### Effect of passive exposure to cigarette smoke on absolute level of systolic blood pressure

Thirteen studies assessed the effect of passive exposure to cigarette smoke on absolute level of systolic blood pressure. After pooling the amounts of adjusted regression coefficients, it was shown that passive exposure to cigarette smoke leads to a significant increase in absolute level of systolic blood pressure (pooled coefficient = 0.26; 95% CI: 0.12 to 0.39) (Fig. 4). Heterogeneity was observed in this section (I2 = 50.4%; *p* = 0.004), but publication bias was not seen (coefficient = 3.98; *p* = 0.06).

Subgroup analysis showed that type of study, different age groups among children, different definitions of smoking, period of exposure and sample size were the most important causes of heterogeneity among studies. Pooled analysis of cohort studies showed that passive exposure to cigarette smoke increases absolute level of systolic blood pressure (*p* < 0.001); however, this association was not seen in cross-sectional studies (*p* = 0.44). Moreover, passive exposure in patients between the ages of 0 and 7 years old (*p* < 0.001) and 12 and 18 years old (*p* = 0.001) was associated with higher levels of systolic blood pressure. In addition, passive exposure to cigarette smoke of individuals who are current daily smokers (*p* = 0.003) or smoke at least one cigarette per week (*p* = 0.003) leads to an increase in absolute level of systolic blood pressure in children. Additionally, exposure to cigarette smoke during fetal period (*p* < 0.001) is also associated with an increase in absolute level of systolic blood pressure in childhood and adolescence (Table 4).

**Table 4:** Subgroup analysis of smoking effects on pediatric systolic blood pressure

Category	Model	Publication bias	Heterogeneity^a^	Beta (95%CI)	P_for effect size_
Age group (year)					
0–7	FEM	*p* = 0.55	33.0% (*p* = 0.11)	0.39 (0.24 to 0.55)	< 0.001
7–13	FEM	*p* = 0.04	0.0% (*p* = 0.86)	0.14 (− 0.12 to 0.40)	0.31
12–18	FEM	*p* = 0.68	53.2% (*p* = 0.12)	0.21 (0.09 to 0.33)	0.001
Type of study					
Cohort	FEM	p = 0.04	27.2% (p = 0.14)	0.25 (0.16 to 0.34)	< 0.001
Cross-sectional	REM	p = 0.68	62.5% (*p* = 0.03)	0.21 (−0.32 to 0.74)	0.44
Smoking definition					
Not reported	FEM	*p* = 0.88	28.8% (*p* = 0.24)	0.16 (−0.38 to 0.70)	0.57
At least 1 cigarette per month	FEM	*p* = 0.47	29.0% (*p* = 0.21)	0.02 (−0.09 to 0.13)	0.71
Current daily smoking	FEM	*p* = 0.83	45.7% (p = 0.14)	0.25 (0.08 to 0.41)	0.003
At least 1 cigarette per month	REM	*p* = 0.82	55.3% (p = 0.004)	0.30 (0.10 to 0.50)	0.003
Period of exposure					
Pregnancy	REM	*p* = 0.02	35.4% (p = 0.08)	0.26 (0.12 to 0.41)	< 0.001
Domestic (postnatal)	REM	p = 0.07	59.8% (p = 0.03)	0.28 (−0.04 to 0.59)	0.08
Parental smoking habit					
Mother	REM	p = 0.04	43.2% (p = 0.04)	0.25 (0.09 to 0.41)	0.002
Father	NA	NA	NA	NA	NA
Both	REM	*p* = 0.05	56.8% (p = 0.03)	0.34 (0.01 to 0.67)	0.04
Sample size					
< 1000 subjects	FEM	*p* = 0.91	27.5% (*p* = 0.25)	0.61 (−0.41 to 1.63)	0.24
≥ 1000 subjects	REM	p = 0.07	54.6% (p = 0.003)	0.25 (0.11 to 0.39)	< 0.001
BP measurement device					
Mercury/aneroid	REM	0.576	56.5% (*p* = 0.011)	0.11 (0.03 to 0.20)	0.007
Automatic/semiautomatic	FEM	0.257	26.7% (*p* = 0.190)	0.33 (0.17 to 0.48)	< 0.001

^a^Heterogeneity was reported as I-squared and corresponding p value. *CI* Confidence interval, *FEM* Fixed effect model, *NA* Not applicable due to lack of included studies, *REM* Random effect mode

### Effect of active smoking on absolute level of diastolic blood pressure

4 studies were entered in this section. Active smoking did not have a significant effect on absolute level of diastolic blood pressure (pooled coefficient = 0.01; 95% CI: -0.18 to 0.20). Heterogeneity was observed in this section (I2 = 51.7%; *p* = 0.08), but publication bias was not seen (coefficient = 1.02; *p* = 0.39). The source of heterogeneity could not be found due to scarcity of studies (Fig. 4).

### Effect of passive exposure to cigarette smoke on absolute level of diastolic blood pressure

6 studies assessed the effect of passive exposure to cigarette smoke on absolute level of diastolic blood pressure. Similar to active smoking, passive exposure to cigarette smoke did not have a significant effect on absolute level of diastolic blood pressure (pooled coefficient = 0.07; 95% CI: -0.15 to 0.29). Heterogeneity was observed in this section (I2 = 83.9%; *p* < 0.001), but publication bias was not seen (coefficient = 4.90; *p* = 0.44). Subgroup analysis could not be done in this section due to scarcity of studies.

## Discussion

For the first time, the present meta-analysis assessed the effect of active smoking or passive exposure to cigarette smoke on risk of developing hypertension in children and adolescents. Although analyses showed that active smoking or passive exposure to cigarette smoke were not associated with developing hypertension in children and adolescents, passive exposure to cigarette smoke was associated with higher levels of systolic blood pressure. In the present study, it was shown that passive exposure to cigarette smoke during fetal period increases the level of systolic blood pressure in childhood and adolescence.

The present meta-analysis showed that active smoking was not associated with developing hypertension or absolute level of blood pressure. The cause of this finding could be found in cumulative effect of cigarette smoking. While assessing cigarette consumption, duration of smoking is an influential factor which should be considered. Hence, the term “pack-year” is used in cigarette studies [57–61]. The mentioned term indicates number of cigarettes used and smoking duration. Adverse effects of cigarette smoking in children and adolescents may not be evident as duration of active smoking is short in this population. There was no study emphasizing on duration of active smoking among entered studies of the present meta-analysis. Therefore, subgroup analysis could not be done based on duration of consumption or exposure.

A longitudinal survey showed that there is no associations between smoking and the risk of hypertension in individuals younger than 35 years old; but smoking was significantly associated with hypertension in older ages [62]. Therefore, it seems that the duration of exposure to cigarette smoke is a potential covariate for assessment of smoking and hypertension. However, most of eligible studies in the current meta-analysis were cross-sectional with short follow-up periods. Therefore, the lack of a significant relationship between smoking and hypertension may be due to limitations of the included studies.

Passive smoking, mainly starting in the fetal period, has a longer duration in children and adolescents than active smoking, which tends to start later on, during adolescence. This issue may be an explanation for the absence of association between active smoking and blood pressure level. Therefore, it is suggested to assess a life-course association of smoking and hypertension in future studies.

Subgroup analysis was done to assess the association between passive exposure to cigarette smoke and absolute level of systolic blood pressure due to presence of significant heterogeneity among related studies. Different definitions of smoking among studies were the most important source of heterogeneity. There was a significant association between passive exposure to cigarette smoke and absolute level of systolic blood pressure in studies which smoking was defined as number of cigarettes smoked per day or week. However, a significant association was not seen in studies which used non-standard definitions such as “smoker or non-smoker”. Overall, definition of smoking was diverse among studies. Therefore, it is possible that some cases are wrongly put in smoker group and hence explaining the non-significant association seen between cigarette smoking and blood pressure.

Effect of cigarette smoking in parents during pregnancy on absolute level of systolic blood pressure in childhood and adolescence was one of the most important findings of the present study. Absolute levels of blood pressure were higher in children who their parents especially their mothers had a history of cigarette smoking. The cause of mentioned finding might be due to the effect of harmful substances present in cigarette smoke on fetal growth [44]. This finding shows that although active or passive exposure to cigarette smoke does not lead to development of hypertension in children and adolescence, it results in higher levels of absolute blood pressure in this age group. The importance of this finding is that elevated level of absolute blood pressure in childhood is a known risk factor for hypertension during adulthood. Hence, these children might get hypertension during adulthood [63–66].

Although blood pressure measurement methods were slightly different among studies, most of them used the standard protocol for BP measurement. Apart from two articles, other studies attempted to measure blood pressure at least 2 times and included the mean of these two values in their analyses. The only major diversity among eligible studies was the device used to measure blood pressure. 11 studies used mercury or aneroid sphygmomanometer devices while 18 studies used automatic oscillometric devices. Subgroup analysis showed that the type of blood pressure measurement device does not affect the relationship between smoking and systolic blood pressure value. Therefore, it seems that the method of measuring blood pressure does not affect the findings of this study.

## Limitations

High level of heterogeneity among studies was one of limitations of the present study. Different definitions of smoking were the most important source of heterogeneity and led to use of random effect analysis in order to present a more conservative effect size. Definition of smoking was not standard in many studies as many studies which were highly focused on cigarette smoking defined smoking as consumption of at least 100 cigarettes [67–69]. However, the mentioned definition was not used in any of entered studies. In many studies cigarette smoking was defined as consumption of at least 1 cigarette per day, but this definition may be biased due to lack of information about duration of smoking. Follow up period was diverse among studies as researchers of the present study could not categorize studies according to their follow-up period for further assessments. Additionally, adjusting for confounders in order to assess reported associations had a high diversity in different studies. Some of them had entered socio-economic and socio-demographic factors in their models while they were not entered in other studies. Therefore, difference in adjustments might be another factor influencing results.

## Conclusion

The present study showed that both active and passive cigarette smoking were not associated with developing hypertension in children and adolescents. However, exposure to passive cigarette smoke was associated with higher level of systolic blood pressure in children and adolescents.

## Abbreviations

BP: Blood pressure
CI: Confidence interval
HTN: Hypertension
NA: Not applicable
NR: Not reported
